# AURKA inhibitor-induced PD-L1 upregulation impairs antitumor immune responses

**DOI:** 10.3389/fimmu.2023.1182601

**Published:** 2023-09-12

**Authors:** Bi Meng, Xuan Zhao, Shuchang Jiang, Zijian Xu, Sijin Li, Xu Wang, Wen Ma, Liantao Li, Dan Liu, Junnian Zheng, Hui Peng, Ming Shi

**Affiliations:** ^1^ Cancer Institute, Xuzhou Medical University, Xuzhou, Jiangsu, China; ^2^ Center of Clinical Oncology, The Affiliated Hospital of Xuzhou Medical University, Xuzhou, Jiangsu, China; ^3^ Jiangsu Center for the Collaboration and Innovation of Cancer Biotherapy, Xuzhou Medical University, Xuzhou, Jiangsu, China; ^4^ Department of Operational Medicine, Tianjin Institute of Environmental & Operational Medicine, Tianjin, China

**Keywords:** targeted drugs, MLN8237, AURKA inhibitor, PD-L1, p-STAT3, tumor immunotherapy

## Abstract

**Introduction:**

Tumor immunotherapy targeting PD-L1 has emerged as one of the powerful tools for tumor therapy. Numerous studies indicate that tumor-targeted drugs critically have an influence on the interaction between the immune system and tumors by changing the expression of PD-L1, which is beneficial for immunotherapy. Our study provided novel evidence for improving the drug regimen in tumor targeted therapy and immunotherapy.

**Methods:**

The expression of PD-L1 on SKBR3, MDA-MB-231, MCF7, 4T1, MC38 and B16 cells was evaluated by flow cytometry after treatment with six preclinical targeted drugs (ARN-509, AZD3514, Galeterone, Neratinib, MLN8237 and LGK974). AURKA was knockdowned by using the specific siRNA or CRISPR-Cas9 technology. In the 4T1-breast tumor and colorectal cancer xenograft tumor models, we determined the number of infiltrated CD3+ and CD8+ T cells in tumor tissues by IHC.

**Results:**

We found that AURKA inhibitor MLN8237 promoted the expression of PD-L1 in a time- and concentration-dependent manner while exerted its antitumor effect. Knockdown of AURKA could induce the upregulation of PD-L1 on SKBR3 cells. MLN8237-induced PD-L1 upregulation was mainly associated with the phosphorylation of STAT3. In the 4T1-breast tumor xenograft model, the infiltrated CD3+ and CD8+ T cells decreased after treatment with MLN8237. When treated with MLN8237 in combination with anti-PD-L1 antibody, the volumes of tumor were significantly reduced and accompanied by increasing the infiltration of CD3+ and CD8+ T cells in colorectal cancer xenograft tumor model.

**Discussion:**

Our data demonstrated that MLN8237 improved the effect of immunology-related therapy on tumor cells by interacting with anti-PD-L1 antibody, which contributed to producing creative sparks for exploring the possible solutions to overcoming drug resistance to tumor targeted therapy.

## Highlights

1. Our findings provided some novel clues to elucidate the poor efficacy of MLN8237 in anti-tumor clinical trials.
2. A new and effective combination regimen was proposed in our research that MLN8237 combined with immune checkpoint inhibitor could improve the effect of tumor therapy.
3. We discovered a new mechanism for the up-regulation of PD-L1, by which tumor cells may evade immunological surveillance when their cell cycle progression was unexpectedly breakdown.

## 1 Introduction

Recent studies have shown that clinical antitumor therapy strategies, including surgery, radiotherapy, chemotherapy and targeted therapy, can trigger immune responses to malignant tumors (1). The immunogenicity of tumor cells plays critical roles in modulating antitumor immune response by inducing the infiltration of CD8^+^ T cells or inhibiting other various immunosuppressive cells in the tumor microenvironment (TME) (2). Recent studies on tumor-targeting drugs reveal that it has a positive influence on the immune system. For instance, anthracycline antibiotics-mediated immunology attenuates immune suppression and enhances the efficacy of immunotherapy (3). The tyrosine kinase inhibitor imatinib promotes the infiltration of NCAM1-secreting NK cells and significantly prolongs the progression-free survival of patients in gastrointestinal stromal cancer patients (4). Therefore, the changes of signaling molecule in the immune microenviroment play a crucial role in poor response induced by clinical treatment.

Immune checkpoint PD-1/PD-L1 pathway can result in tumor immune escape (5). PD-L1 expressed on the surface of tumor cells binds to PD-1 expressed on the surface of immune cells, and then phosphorylates the tyrosine motif of the immune receptor domain in the intracellular domain of PD-1, thereby releasing inhibitory signals, promoting T cell apoptosis and depletion, and inhibiting T cell proliferation. Moreover, PD-1 phosphorylation weakens the killing effect of T cells and leads to tumor immune escape through enhancing the function of immunosuppressive Treg cells, inhibiting tumor antigen presentation and the function of effector T cells, or protecting tumor cells from the CD8^+^ T cell-mediated phagocytosis (5–8). Therefore, immunotherapy for PD-L1 is of vital importance to tumor therapy. Immune checkpoint inhibitor therapy targeting PD-L1 has been approved by the Food and Drug Administration (FDA) of USA for clinical applications (9–11). A growing body of research indicates that PD-L1 is highly expressed on certain cancer cells, including cervical cancer (CESC), lymphoma, thymic cancer and so on (12–14). The expression of PD-L1 is regulated by various factors, including internal and external factors (15). For instance, the level of PD-L1 is remarkably increased in response to the enhancement of 9p24.1 chromosome copy number in Hodgkin’s lymphoma (16). Some studies identify that epigenetic modifications, such as DNA methylation, can inhibit the expression of PD-L1 in primary breast cancer (PBC) and colorectal cancer (CRC) (17). In addition, histone deacetylase HDAC8 is already known to participate in the inhibition of the transcriptional activation of PD-L1 in melanoma cells (18). Therefore, the inhibition of HDAC8 may be advantageous for increasing the expression of PD-L1. In the complex tumor microenvironment, PD-L1 can also be upregulated under the stimulation of different signaling pathways induced by inflammatory factors, such as gamma-interferon (IFN-γ), epidermal growth factor (EGF), and IL-17 (19).

Multiple targeted drugs have been reported to be associated with the upregulation of PD-L1. Pharmaceutical inhibition of CDK4/6 targeted cyclin-dependent kinases (CDKs) upregulates the protein level of PD-L1 through inhibiting CDK4-mediated SPOP phosphorylation at Ser6 and promoting the degradation of SPOP by E3 ubiquitin ligase complex APC/C^Cdh1^. Recent studies also demonstrate that targeting CDK4 can significantly stabilize PD-L1 through promoting the degradation of SPOP, which implicating the possibility for combination therapy of CDK4/6 inhibitors and PD-1/PD-L1 immune checkpoint inhibitors (20). Further development of ATR kinase inhibitors (ATRi) has demonstrated that it can abolish G2-M cell cycle checkpoint (G2-M block) regulated by ATR-checkpoint kinase 1 (CHK1)-CDK1 and result in the death of prostate cancer cells. ATRi combined with anti-PD-1 antibodies can efficiently enhance T cell-mediated tumor cell death. ATR suppression therapy combined with immune checkpoint blockades has been in early clinical trials in tumors, such as prostate cancer (NCT04266912 and NCT04095273) (21). PARPi (PARP inhibitors) remarkably increases the level of PD-L1 by impairing hyperphosphorylation of glycogen synthase kinase 3β (GSK3β) (p-Ser9), which subsequently inhibits the antitumor immunity in both breast cancer cells and animal models. The blockade of PD-L1 expression contributes to enhancing the efficacy of PARPi in cancer treatments by activating the killing activity of T cells. In addition, PARPi and ATRi, which have a critical role in anti-cancer treatment, are regulators of immunotherapy and primarily act on biliary tract carcinoma (BTC) (22–24). Therefore, the overexpression of PD-L1 induced by targeted drug therapy is crucial for the regulation of antitumor immunity. However, the mechanism of targeted drugs induced PD-L1 overexpression has not yet been fully identified.

In this study, we revealed that AURKA inhibitor MLN8237 upregulated the expression of PD-L1 in breast cancer by increasing the level of p-STAT3 and impeding the infiltration of CD3^+^ T and CD8^+^ T cells in tumor tissues, thus impaired antitumor immune response. The combination of MLN8237 and anti-PD-L1 antibody effectively inhibited the progression of colon cancer. Therefore, we provided a new strategy for MLN8237 combined with immune checkpoint inhibitors to improve the immune response and treatment effect of colon cancer.

## 2 Materials and methods

### 2.1 Cell lines

Human breast cancer cell line SKBR3, MDA-MB-231 and MCF-7 cells were obtained from ATCC (American Type Culture Collection) and preserved in the Immunology Laboratory of Jiangsu Cancer Institute. Mouse breast cancer cell line 4T1, mouse colon cancer cell MC38 and mouse melanoma cell B16 were from the Academy of Military Medical Sciences and preserved in our laboratory. CRISPR/Cas9 engineered 293T cells, stably expressing Cas9, were purchased from ATCC (American Type Culture Collection).

### 2.2 Reagents

DMEM medium, fetal bovine serum, T4 ligase, T7 endonuclease, Escherichia coli DH5α, plasmid extraction kit, genomic DNA extraction kit, reverse transcription kit, and SYBR^®^ Green RT-PCR Master Mix were purchased from TransGen Biotech Co (Beijing). ACCUTASE™ cell digestion solution was purchased from eBioScience Inc. Western blot antibodies, STAT1 (14994), p-STAT1 (Tyr701, 7649P). STAT3 (9132), p-STAT3 (Tyr705, 9145), STAT5 (9363p), p-STAT5 (Y694, 4322). AURKA (14475), p-AURKA (Thr288, 3079), PD-L1 antibody (13684), p-AKT (Ser473, 4060), AKT (pan) (4691), p-ERK/ERK antibody (4370), GAPDH (5174), β-tubulin (2128) were purchased from Cell Signaling Technology, CD3 histochemical antibody (100219), CD8 histochemical antibody (104705) were purchased from Biolegend, and 4% paraformaldehyde were purchased from Wuhan Seville Biological LTD. DAB histochemical staining kit was purchased from Zhongshan Jinqiao Biological Technology LTD (Beijing). ECL luminescence color reagent was purchased from Healthcare. Trizol RNA extraction reagent was purchased from Sigma-Aldrich (T9424). Anti-mouse PD-L1 Antibody was purchased from BioXcell.

### 2.3 Knocking down the expression of AURKA in breast cancer cell SKBR3

The single U6 sgRNA primer was designed and synthesized. According to the CRISPR/Cas9 design principle, six sgRNAs for *AURKA* gene, complementary to the sticky ends formed by the pGL3-U6-sgRNA-PGK-Puro expression plasmid vector after digestion with BsaI were designed. The designed sgRNA oligonucleotide sequence is shown in **Table 1-1**. To construct plasmids containing the six sgRNA sequences, the PCR product was recovered by using a DNA Clean up Kit (TransGen Biotech Co, Beijing) and then was ligated into the BsaI linearized pGL3-U6-sgRNA-PGK-Puro plasmid with T4 DNA ligase. The recombinant plasmid was used to transform competent cells and the positive clones were cultured, plasmid purified and sequenced (Genewiz, China). The genomic DNA of CRISPR/Cas9 engineered 293T cells seeded in a 12-well plate at 1.8×10^5^/well was extracted and amplified by PCR. PCR products were digested with T7 restriction enzymes. The graph was taken and the amount of DNA was analyzed using a grayscale-based method to determine the cutting efficiency (**Supplement Figure 3**). The calculation formula is as follows: Cutting efficiency (%) = (addition of the gray-scale scanning results of the following two cutting bands/gray-scale scanning results of the full-length bands) × 100%. A pair of sgRNA with higher cutting efficiency (namely sgRNA 1 and sgRNA 4) was selected to design *AURKA* targeting 2U6 sgRNA primers. The designed oligonucleotide sequences of *AURKA* 2U6 sgRNA primer are shown in **Table 1-2**. The synthesized primer sequence was ligated into the PUC57-U6-sgRNA vector and a double strand DNA sequence was amplified and ligated into an Esp3I (BsmBI) restriction endonuclease predigested pGL3-U6-sgRNA-ccdB-EF1α-Puro vector using T4 DNA ligase.

**Table 1-1:** sgRNA Oligonucleotide sequence targeting *AURKA* gene.

SgRNA	Forward Primer	Reverse Primer
sgRNA 1 sgRNA 2 sgRAN 3 sgRNA 4 sgRNA 5 sgRAN 6	5’-accgatccattacctgtaaatag-3’ 5’-accgttacctgtaaatagtggcc-3’ 5’-accgcttgtctccagtcacaagc-3’ 5’-accgcacgttttggacctccaac-3’ 5’-accggagtcacgagaacacgttt-3’ 5’-accgtgcgctgggaagaatttga-3’	5’-aaacctatttacaggtaatggat-3’ 5’-aaacggccactatttacaggtaa-3’ 5’-aaacgcttgtgactggagacaag-3’ 5’-aaacgttggaggtccaaaacgtg-3’ 5’-aaacaaacgtgttctcgtgactc-3’ 5’-aaactcaaattcttcccagcgca-3’

**Table 1-2:** AURKA targeting 2U6 sgRNA primer oligonucleotide sequence.

Primer	Forward	atgcgtctcaaccgcacgttttggacctccaacgttttagagctagaaatagcaag
	Reverse	atgcgtctcgaaacctatttacaggtaatggatcggtgtttcgtcctttccacaag

**Table 1-3:** PCR Primersh represent human.

Gene	Forward	Forward and Reverse Primer
*hPD-L1*	Forward Primer Reverse Primer	5’-TCACGGTTCCCAAGGACCTA-3’ 5’-AGAGCTGGTCCTTCAACAGC-3’
*GAPDH*	Forward Primer Reverse Primer	5’-CAAGGTCATCCATGACAACTTTG-3’ 5’-GTCCACCACCCTGTTGCTGTAG-3’

### 2.4 Transfection

The correctly ligated plasmid was used to transfect SKBR3 cells. Briefly, SKBR3 cells were seeded in a 6-well plate at a density of 4.5×10^5^. 2U6 plasmid and Cas9 plasmid were each mixed with Lipofectamine 2000 transfection reagent at a ratio of 1:2.5 and then dripped evenly into the culture plate. After being cultured for 24 h, puromycin was added into the culture medium at a concentration of 1 μg/ml for selecting positive clones. Cells transfected with or without Cas9 plasmid were used as positive and negative controls, respectively.

### 2.5 Western blot

The protein was extracted and its concentration was determined by using a BCA protein concentration determination kit. The whole-cell lysates were separated by SDS-PAGE. Extracted protein were resolved by SDS-PAGE electrophoresis, and transferred to nitrocellulose filter membrane (NC). The membrane was blocked with Tris-buffered saline containing 0.1% Tween-20 (1×TBST) and 5% skimmed milk by incubating at room temperature for 60 min and washed with 1×TBST. The membrane was incubated with primary antibody (1:1000) dissolved in 1×PBS containing 5% BSA overnight at 4°C. After the incubation, the membrane was washed with 1×TBST and then incubated with secondary antibodies (1:5000) diluted in 1×TBST containing 5% skimmed milk. The membrane was further incubated at room temperature for 1 h, and washed with 1×TBST for three times. The target protein bands were visualized and recorded with a chemiluminescence imaging system (Tanon) using the Enhanced Chemiluminescence System (Amersham Pharmacia Biotech).

### 2.6 Quantitative real-time PCR

Total RNA extraction: 1 ml Trizol was added to each well to lyze the cells. Then 200 μl of chloroform was added, followed with vigorous shaking, incubation at room temperature for 10 min, and centrifugation at 12,000 g for 15 min at 4°C. The upper colorless liquid was transferred into a 1.5 ml EP tube and then the same volume of isopropanol was added. After centrifuging at 12,000 g for 15 min at 4°C, the supernatant was discarded. The precipitated RNA was washed with 1 ml of 75% ethanol (diluted with DEPC water) and finally dissolved into DEPC water. Reverse transcription: reverse transcription of the extracted mRNA into cDNA was performed by using a reverse transcription kit in accordance with the manufacturer’s instructions. A real-time quantitative PCR was used to analyse the expression level of the target gene through the formula 2^-△△Ct^. GAPDH was used as an internal reference. All primers were synthesized by Suzhou Genewiz Biotechnology LTD, and the primer sequences are shown in **Table 1-3**.

### 2.7 Flow cytometry

After 48 h of drug stimulation, the cells were rigorously washed by 1×PBS and digested with ACCUTASE™ cell digestion solution, neutralized with DMEM medium containing 5% fetal calf serum, centrifuged at 1000 rpm for 5 min, and resuspended in 100 μl 1×PBS. Afterwards, the resuspended cells were labeled with PE-conjugated PD-L1 by incubating at room temperature for 30 min in the dark. After washed with 1×PBS, the cell samples were then examined by flow cytometry. Based on the detection principle of flow cytometry, the cells to be detected are made into single-cell suspension after being fluorescently labeled. Under the irradiation of laser, the fluorescently stained cells generate scattered light and excited fluorescence. As well as labeled information about the particles inside the measured cell, these light signals are converted into electrical signals, which are transmitted to a computer and converted into data files that are stored and processed. After the data is processed, fluorescence intensity of the labeled protein can be recorded. Then we analyzed the data by using flow cytometry software FlowJo (Flow cell analysis software). According to the analysis results, the fluorescence intensity of PD-L1 on the cells was obtained.

### 2.8 *In vivo* experiments

6-8 weeks old female BALB/c mice and C57BL/6J mice purchased from Beijing Vital River Laboratory Animal Technology Co. Ltd. Mice (n=20) were divided into four groups and each group contained 5 mice. MC38 cells were inoculated subcutaneously in C57BL/6J mice with 3×10^5^ cells per mouse. 4T1 cells were inoculated subcutaneously in BALB/c mice with 3×10^5^ cells per mouse. When the volume of tumor reached to about 30 mm^3^, the administration was started. MLN8237 was dissolved in a mixture of 10% 2-hydroxypropyl-β-cyclodextrin + 1% sodium bicarbonate, and was administered at 15 mg/kg per day based on the body weight of the mouse. Mice were treated with MLN8237 intragastrically for 5 consecutive days. PD-L1 antibody was diluted with 1×PBS and administered intraperitoneally at 200 μg/100 μl per mouse on day 8, 11, and 14. After the administration, the volume of the tumor mass was measured at day 5, 7, 10, 13, 15, and 17, and primary tumors of the mice at day 17 after the administration were dissected, weighed, and fixed in formalin.

### 2.9 Immunohistochemical staining

Tumor samples from the mice were stripped and sent to the company for sectioning. The paraffin sections were dewaxed and hydrated overnight in an oven at 60°C. Immerse the sections sequentially as follows: xylene I and xylene II for 5 minutes each; absolute ethanol I, absolute ethanol II, 95% ethanol, 90% ethanol, 85% ethanol, 80% ethanol, 70% ethanol, and distilled water for 5 minutes each to ensure complete deparaffinization and hydration (step 2). Cross-linking of proteins may occur after tissue has been fixed with formaldehyde, resulting in the loss of antigen and the blocking of antigen-antibody binding. Therefore, antigen recovery processing is required in order for antibodies to bind efficiently to the target antigen. For performing antigen retrieval, place the sections into sodium citrate-Hydrochloric acid Buffer solution (0.01 mol/L, pH 6.0), then heated and boiled for 20 min, cool to room temperature naturally. Incubate in 0.3% Triton X-100 at room temperature for 20 min, away from light, washed with 1×PBS for three times. Add 1% BSA/PBS blocking solution onto the tissue and incubate in a humid chamber at 37°C for 40 minutes. Dilute the primary antibodies of CD3 and CD8 in 1% BSA/PBS at a ratio of 1:100. Add the antibody solution to cover the tissue sections, place in a humid chamber overnight at 4°C. Wash the sections with 1×PBS for three times, remove excess 1×PBS. Tumor tissues were treated with 3% H_2_O_2_ and cultured for 10 minutes in a damp room away from light to remove non-specific background staining caused by endogenous peroxidase. Remove the H_2_O_2_ from the sections, rinse with 1×PBS three times for 5 minutes each. Add goat anti-rat IgG/HRP polymer to fully cover the sections. Place the sections back in the humid chamber and incubate at 37°C for 45 minutes. DAB staining: wash the tissue sections with 1×PBS three times for 5 minutes each. According to the instructions of the DAB kit, mix 50 μl of reagent B into 1 ml of reagent A thoroughly and place it at room temperature, protected from light. Remove excess 1×PBS from the sections and add the freshly prepared DAB reagent to completely cover the tumor tissue. Incubate in a humid chamber at room temperature, protected from light, for 9 minutes. Hematoxylin counterstaining: rinse the sections with distilled water to remove excess DAB solution, shake off the excess water, and immerse the sections in hematoxylin staining solution for 50 seconds. Dehydrate the sections in the reverse order as described in step 2, mount with neutral mounting medium, observe under an optical microscope, and take photographs (20×).

## 3 Results

### 3.1 AURKA kinase inhibitor MLN8237 up-regulated PD-L1 expression

**Figure 1:**
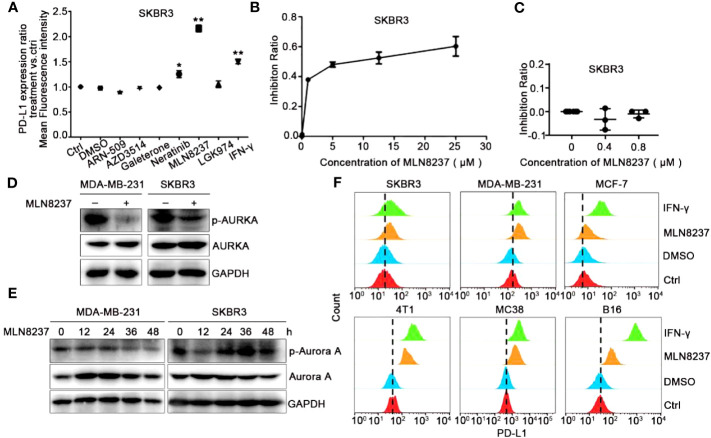
AURKA inhibitor MLN8237 could upregulate the expression of PD-L1 on several different cancer cells. **(A)** Plot of the ratio of PD-L1 expression on SKBR3 cells treated with various inhibitors for 48 h, IFN-γ was used as positive control. **(B, C)** The proliferation curve of SKBR3 cells after treatment with different concentrations of MLN8237. **(D)** SKBR3 or MDA-MB-231 cells were treated with MLN8237 (0.8 μM) and harvested for western blot analysis of p-AURKA/AURKA expression. **(E)** SKBR3 or MDA-MB-231 cells were treated with MLN8237 (0.8 μM) for various times and harvested for western blot analysis of p-AURKA/AURKA expression. **(F)** The levels of PD-L1 on SKBR3, MDA-MB-231, MCF-7, 4T1, MC38, and B16 cells was determined by flow cytometry (FCM) after treatment with MLN8237 at 0.8 μM. IFN-γ (100 ng/ml) was used as the positive control. *P<0.05, **P<0.01.

The half maximal inhibitory concentrations (IC_50_) of SKBR3 cells were detected by CCK-8 in response to stimulation of six drugs (ARN-509, AZD3514 and Galeterone target androgen receptor, Neratinib targets HER2, MLN8237 targets AURKA, and LGK974 targets PORCN), which have been evaluated clinical responses in multiple preclinical studies and clinical trials for different patients (**Supplement Figure 1**). Various strategies of target drugs screens have been utilized to identify and validate novel target drugs, which may be suitable for immunotherapeutic intervention. Therefore, we detected the expression of PD-L1 on the cell membrane by using flow cytometry after incubation with these drugs. As shown in **Figure 1A**, after 48 h of SKBR3 cells treated with various inhibitors, both HER2 inhibitor Neratinib and AURKA inhibitor MLN8237 up-regulated the expression of PD-L1 in SKBR3, while other inhibitors had no significant effect on the expression of PD-L1. Among them, the up-regulation effect of MLN8237 on PD-L1 expression was more than 2 times, which was significantly stronger than the effect of IFN-γ (positive control) on PD-L1 expression, and the difference was statistically significant.

Additionally, we assumed that the structure of MLN8237 might contain some luminescent groups. Flow cytometry was used to rule out the effect of the false positive after stimulation with MLN8237 at 0.8 μM for 48 h in SKBR3 cells. As shown in **Supplement Figure 2**, after MLN8237-treated cells were incubated with PD-L1 antibody, there was no significant shift in flow cytometry results compared with cells that were not treated with MLN8237. Therefore, MLN8237 structure did not contain any luminescent groups, which could exclude the false positive results of the above drug sieves. **Figures 1B, C** indicated that the selected concentration (0.8 μM) of MLN8237 was moderate and much lower than its 50% lethal concentration. Western blot analysis in **Figure 1D** also demonstrated that MLN8237 could remarkably suppress p-AURKA in two breast cancer cell lines. The results in **Figure 1E** showed that the inhibitory effect of MLN8237 on p-AURKA in MDA-MB-231 cells was time-dependent. In SKBR3 cells, there was a tendency in the level of PD-L1 to be activated first and then to be inhibited, implying that the mechanism of MLN8237-induced PD-L1 upregulation might be different in MDA-MB-231 and SKBR3 breast cancer cells. In order to prove whether the upregulation of PD-L1 induced by MLN8237 was generalized, we also detected the expression of PD-L1 on MCF-7, 4T1, MC38 and B16 cells. **Figure 1F** demonstrated that the level of PD-L1 on these cancer cell lines was significantly upregulated after treatment with MLN8237. Therefore, the upregulation effect of MLN8237 on PD-L1 was occurred frequently in various cancer cell lines.

### 3.2 MLN8237 up-regulated PD-L1 expression in a dose- and time-dependent manner

**Figure 2:**
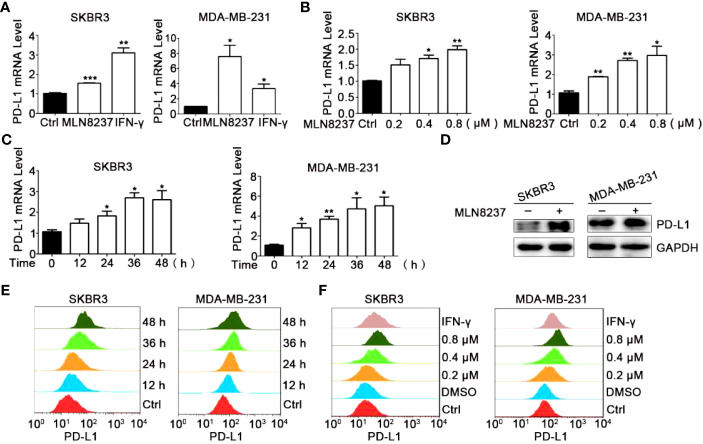
MLN8237 upregulated the expression of PD-L1 on SKBR3 and MDA-MB-231 cells. **(A)** SKBR3 or MDA-MB-231 cells were treated with 0.8 μM MLN8237 for 48 h and harvested for qPCR analysis of *PD-L1* expression. **(B)** SKBR3 or MDA-MB-231 cells were treated with indicated doses of MLN8237 for 48 h and harvested for qPCR analysis of *PD-L1* expression. **(C)** SKBR3 or MDA-MB-231 cells were treated with 0.8 μM MLN8237 for various times and then harvested for qPCR analysis of *PD-L1*. **(D)** SKBR3 and MDA-MB-231 cells were treated with MLN8237 (0.8 μM) for 48 h and harvested for western blot analysis of PD-L1 expression. **(E)** SKBR3 or MDA-MB-231 cells were treated with MLN8237 (0.8 μM) for various times followed by FCM analysis of PD-L1 expression. **(F)** SKBR3 or MDA-MB-231 cells were treated with various doses of MLN8237 for 48 h followed by FCM analysis of PD-L1 expression. *P<0.05, **P<0.01, ***P<0.001.

As shown in **Figure 2**, under the treatment with MLN8237, the up-regulation of PD-L1 mRNA levels in breast cancer SKBR3 and MDA-MB-231 cells exhibited a dose- and a time-dependent manner (**Figures 2A–C**), respectively, consistent with the western blot and flow cytometry results (**Figures 2D–F**). MLN8237 resulted in a dramatic elevation in the protein levels of PD-L1 in breast cancer SKBR3 and MDA-MB-231 cells (**Figures 2D–F**). The up-regulated expression of PD-L1 induced by MLN8237 was detected in a concentration-dependent manner in the range from 0.2 to 0.8 μM. It indicated that the up-regulation of PD-L1 was observed after stimulation with MLN8237 for 12 h in breast cancer cells, and persisted until 48 h.

Above results showed that the level of PD-L1 on the breast cancer cells was evidently upregulated in the group of MLN8237 treatment, whereas some experiments were carried out to completely reverse the effect of MLN8237.

### 3.3 AURKA knockdown in SKBR3 breast cancer cells up-regulated PD-L1 expression

**Figure 3:**
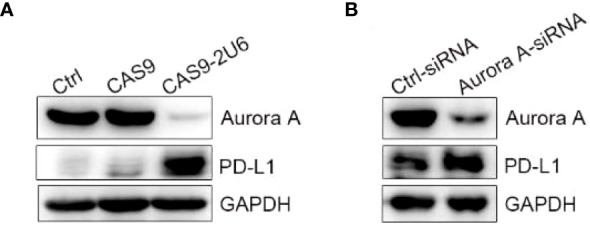
Knock down of AURKA upregulated the expression of PD-L1 in SKBR3 cells. **(A)** SKBR3 was transfected with Cas9-sgRNA1-sgRNA4 plasmid. PD-L1 positive cells were obtained after 5 days of puromycin (1 μg/ml) screening. **(B)** SKBR3 cells treated with siRNA were examined for the expression of PD-L1 by western blot.

In order to explore the effect of AURKA kinase on the up-regulation of PD-L1 by MLN8237, we constructed AURKA kinase knockdown SKBR3 cells by using CRISPR-Cas9 and siRNA. Then western blot was used to confirm the knockdown efficiency of AURKA. Moreover, after AURKA was knocked down, the protein level of PD-L1 increased simultaneously (**Figure 3**).

### 3.4 Inhibition of AURKA expression contributed to the activation of JAK/STAT3 signaling pathway in a dose- and time-dependent manner

**Figure 4:**
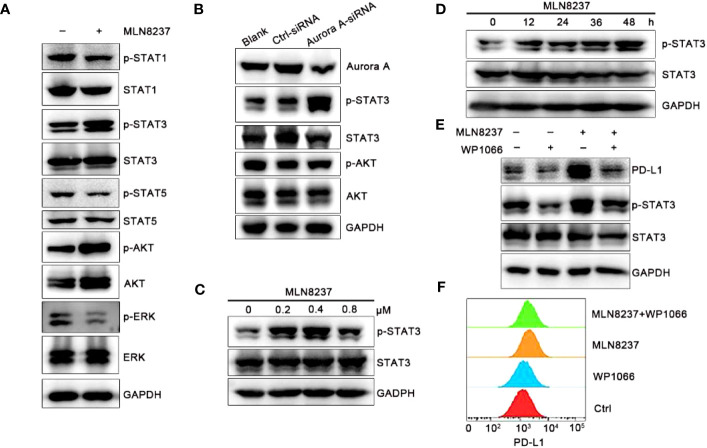
MLN8237 increased the level of p-STAT3 in SKBR3 cells. **(A)** SKBR3 cells were treated with MLN8237 (0.8 μM) for 48 h and harvested for western blot analysis of p-STAT1/STAT1, p-STAT3/STAT3, p-STAT5/STAT5, p-AKT/AKT and p-ERK/ERK. **(B)** The levels of p-STAT3/STAT3 and p-AKT/AKT were determined by western blot after knocking down the expression of AURKA. **(C)** SKBR3 cells were treated with various doses of MLN8237 for 48 h and harvested for western blot analysis of p-STAT3 and STAT3. **(D)** SKBR3 were treated with MLN8237 (0.8 μM) for 48 h, and harvested for western blot analysis of p-STAT3/STAT3 expression. **(E)** SKBR3 cells were treated with MLN8237 (0.8 μM) in the absence or presence of WP1066 (5 μM) for 48 h Western blot analysis of p-STAT3, STAT3 and PD-L1. **(F)** SKBR3 cells were treated with MLN8237 (0.8 μM) in the absence or presence of WP1066 (5 μM) for 48 h followed by FCM analysis of PD-L1.

Recent studies show that the activation of PI3K/AKT signaling pathway promotes the development of tumor resistance in breast cancer (25). The increase of phosphorylated STAT in JAK/STAT signaling pathway is beneficial to the maintenance of tumor therapy resistance (26). The western blot results in **Figure 4A** showed that after MLN8237 stimulation, p-STAT3 and p-AKT were both significantly upregulated, while p-STAT1, p-STAT5, and p-ERK were significantly down-regulated. The results suggested that JAK/STAT3 or PI3K/AKT (phosphatidylinositol 3-kinase/Protein kinase B) signaling pathway may be involved in MLN8237-mediated increase of PD-L1 expression. We found that after AURKA knockdown, the expression of p-STAT3 was enhanced, but the expression of p-AKT did not change significantly (**Figure 4A**). The results showed in **Figure 4B** suggested that the upregulation of PD-L1 caused by MLN8237 may be related to the activation of the JAK/STAT3 signaling pathway.

The JAK/STAT3 signaling cascade is an important signaling pathway in mammals, which can induce cell proliferation and differentiation by affecting the secretion of cytokines and growth factors (27). However, in human tumor cells, abnormal activation of the JAK/STAT3 signaling pathway occurs frequently, affecting the survival and proliferation of tumor cells, and is closely related to the poor prognosis of tumor treatment (28). Therefore, we next mainly studied the role of STAT3 in the upregulation of PD-L1 induced by MLN8237.

SKBR3 cells were stimulated with MLN8237 at 0.2 μM, 0.4 μM, and 0.8 μM for 48 h, and protein at different drug concentrations was extracted to detect the expression level of p-STAT3/STAT3. SKBR3 cells were treated with MLN8237 at 0.8 μM for 12, 24, 36 and 48 h, and then protein was extracted to detect the expression level of p-STAT3/STAT3. Western blot results in **Figures 4C, D** indicated that the upregulation of p-STAT3 caused by MLN8237 exhibited a time- and dose-dependent manner. The dosing experiment showed that the upregulation of p-STAT3 existed between 0.2-0.8 μM, and the up-regulation was strongest at the concentration of 0.4 μM. The time-course experiment results were consistent with the above results in that the expression of PD-L1 gradually increased with the increasing time. **Figure 4E** showed that WP1066 could significantly inhibit the expression of p-STAT3. Moreover, WP1066 and MLN8237 in combination significantly inhibited the up-regulation of PD-L1 and p-STAT3 caused by MLN8237. The expression of PD-L1 under the same conditions was detected by flow cytometry and the results were consistent with those of western blot, suggesting that MLN8237 upregulated the expression of PD-L1 by activating STAT3 (**Figure 4F**).

### 3.5 MLN8237 inhibited the infiltration of CD8^+^ T cells in 4T1 xenograft tumors

**Figure 5:**
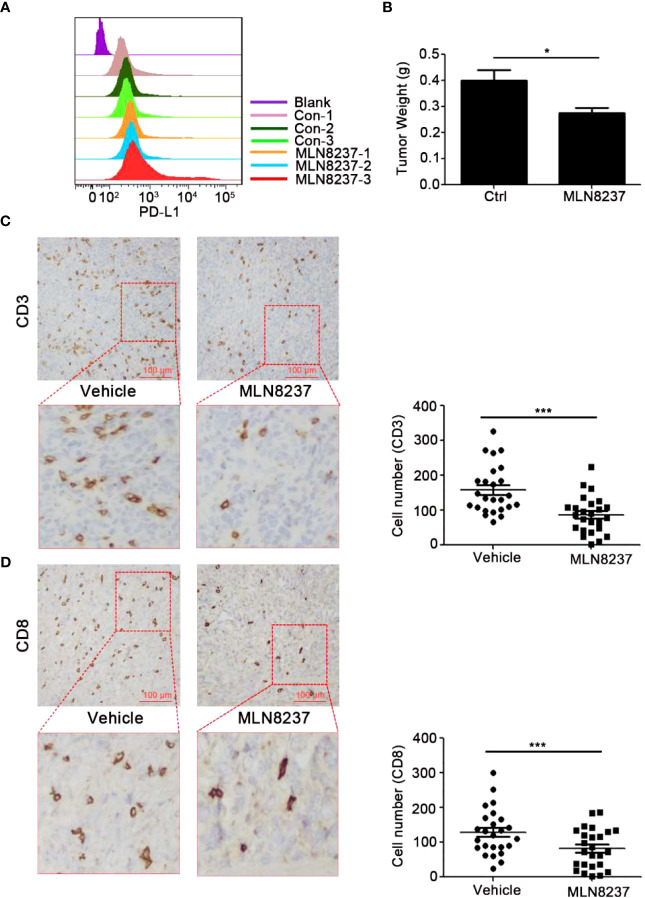
MLN8237 upregulated the expression of PD-L1 and inhibited the infiltrations of CD3^+^ and CD8^+^ T cells *in vivo* with 4T1 mouse breast cancer cells. **(A)** 4T1 cells were inoculated subcutaneously in BALB/c mice with 3×10^5^ cells per mouse, and the day of cells inoculation was the day 0. When the transplanted tumor volumes reached about 30 mm^3^, the administration was started. MLN8237 was dissolved in a mixture of 10% 2-hydroxypropyl-β-cyclodextrin + 1% sodium bicarbonate, and was administered at 15 mg/kg per day based on the body weight of the mouse. Mice were given intragastrically for 5 consecutive days. 17 days after the administration, the tumor was collected and was detected by FCM analysis of PD-L1 expression. **(B)** 4T1 cells were inoculated subcutaneously in BALB/c mice 17 days after the administration, the tumor mass was weighed. **(C, D)** Paraffin sections and immunohistochemical stainings were performed on the transplanted tumor tissues in each group (5 mice), and 5 visual fields were photographed for each immunohistochemical section. Statistical analysis of the number of CD3^+^ and CD8^+^ T cells in 25 different visual fields. *P<0.05, ***P<0.001.

**Figure 6:**
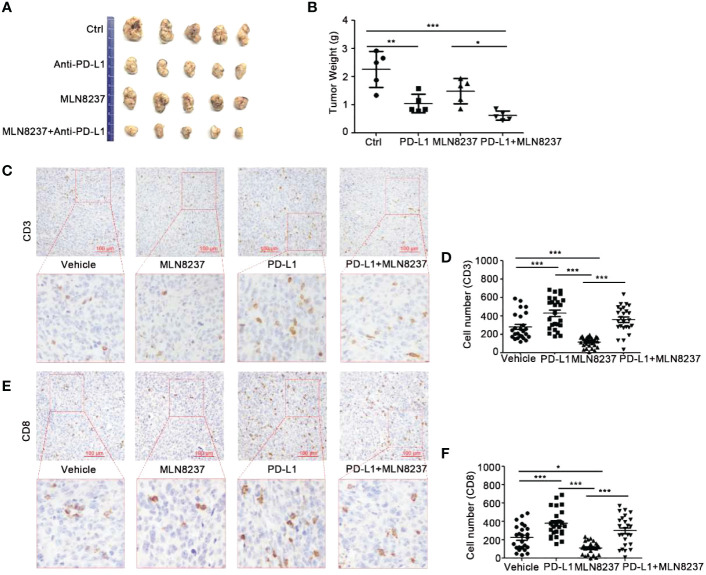
The combination of MLN8237 and anti-PD-L1 antibody could effectively inhibit tumor growth and increase the infiltration of CD3^+^ and CD8^+^ T cells *in vivo.* **(A, B)** C57BL/6J mice were divided into 4 groups and inoculated with MC38 cells. When the transplanted tumor volumes reached about 30 mm^3^, Mice in the four groups were then administered with solvent, anti-PD-L1 antibody, MLN8237, anti-PD-L1 antibody + MLN8237, respectively. MLN8237 was dissolved in a mixture of 10% 2-hydroxypropyl-β-cyclodextrin + 1% sodium bicarbonate, and was administered at 15 mg/kg per day based on the body weight of the mouse. Mice were given intragastrically for 5 consecutive days. PD-L1 antibody was diluted with PBS and administered intraperitoneally at 200 μg/100 μl per mouse on day 8, 11, and 14. 17 days after the administration, the tumor mass was collected, photographed, and weighed. **(C–F)** Paraffin sections and immunohistochemical stainings were performed on the transplanted tumor tissues in each group (5 mice), and 5 visual fields were photographed for each immunohistochemical section. Statistical analysis of the number of CD3^+^ and CD8^+^ T cells in 25 different visual fields. *P<0.05, **P<0.01, ***P<0.001.

4T1 cells were inoculated subcutaneously in BALB/c mice (**Figure 5**), and C57BL/6J mice were divided into four groups and inoculated with MC38 xenograft tumors (**Figure 6**). Mice in the four groups were then administered with solvent, MLN8237, anti-PD-L1 antibody, and a combination of medications, respectively. 17 days after the administration, the tumor mass was collected, photographed, and weighed. Tumor sections were prepared and immunohistochemical staining was used to detect the number of CD3^+^ T cells and CD8^+^ T cells in each group of tumor tissues.

### 3.6 MLN8237 combined with anti-PD-L1 antibody significantly inhibited the growth of MC38 xenograft tumors

The administration of anti-PD-L1 antibody significantly inhibited tumor growth. When MLN8237 was combined with anti-PD-L1 antibody, significantly tumor growth inhibition was observed. Moreover, with the prolongation of the administration time, the inhibitory effect of the two-drug regimen group showed more obviously, and the data was statistically significant (**Figures 6A, B**).

As shown in **Figure 6C**, the immunohistochemical staining results demonstrated that the infiltration of CD3^+^ T cells in the tumors treated with MLN8237 decreased, while it was increased in the anti-PD-L1 antibody group. Moreover, anti-PD-L1 antibody could reverse MLN8237-induced decrease in CD3^+^ T cells infiltration. **Figure 6E** also showed that the number of CD8^+^ T cells in the tumor tissue of the MLN8237 group decreased, and the number of CD8^+^ T cells in the anti-PD-L1 antibody group increased significantly. The number of CD8^+^ T cells in tumor tissues in the combination group (MLN8237 and anti-PD-L1 antibody) was more than that in the MLN8237 group and less than that in the anti-PD-L1 antibody group. The data was statistically significant.

The above results indicated that MLN8237 inhibited CD8^+^ T cell infiltration in tumor tissues, and anti-PD-L1 antibody could reverse the decrease of CD8^+^ T cell infiltration in tumor tissues induced by MLN8237 therapy.

## 4 Discussion

Previous studies demonstrate that there is an abnormal high expression and activation of AURKA in breast cancer (29–31). A substantial number of AURKA inhibitors have been currently under investigation in clinical trials. MLN8237, also named Alisertib, is a selective inhibitor of AURKA. It can disrupt mitosis and cause a remarkable increase in cell death (32). A phase III pilot clinical study employing MLN8237 has recently been initiated as a separate category of drug in the lymphoid lymphoma. Adverse reactions caused by MLN8237 are the main reasons for the termination of the first randomized Phase III clinical trial (NCT01482962). Further research is required to ensure the antitumor effects of MLN8237 therapy and relieve the toxic adverse effects associated with the clinically treatment.

The AURKA inhibitor MLN8237 is currently being studied in clinical trials, but it has not achieved satisfactory efficacy. Intensively exploring the biological effects of MLN8237 on tumor can contribute to developing more reasonable combination treatment strategies (33). In this study, we found that MLN8237 enhanced PD-L1 expression on the surface of breast cancer cells. *In vivo*, we observed that MLN8237 upregulates the expression of PD-L1 and reduces the infiltration of CD8^+^ T cells in tumor tissue. These results suggest that MLN8237 may inhibit the anti-tumor immune function of T cells by upregulating PD-L1. Therefore, we chose to combine MLN8237 with anti-PD-L1 antibodies to treat tumors. Our experimental results (**Figures 5**, **6**) also proved that MLN8237 combined with anti-PD-L1 antibody can enhance the efficacy of tumor therapy. The data in clinical study (NCT01639911) demonstrate that MLN8237 and the tyrosine kinase inhibitors of vascular endothelial growth factor receptor (VEGFR) can partially alleviate the symptom of patients with breast cancer (34). The co-targeting blockade of AURKA and the PI3K signaling pathway is available in the breast cancer models. The combination of them allows patients to tolerate side effects and increase the antitumor effect of MLN8237 (35).

Additionally, there is growing evidence that changes in breast cancer patients’ immune systems can affect how they respond to standard treatments (36). In this study, we found that MLN8237 enhanced the expression of PD-L1 on the surface of breast cancer cells. *In vivo*, MLN8237 upregulated PD-L1 and reduced the infiltration of CD8^+^ T cells in the tumor tissues. It suggested that MLN8237 may suppress the anti-tumor immune function of T cells by upregulating PD-L1. Therefore, anti-PD-L1 antibody was chosed to combine with MLN8237 for anti-tumor treatment. Our experimental results (**Figures 5**, **6**) supported the above hypothesis. Therefore, the combination of MLN8237 and anti-PD-L1 antibody may enhance the effectiveness of anti-tumor treatment. The combination of targeted drugs and immunization can improve the efficacy of immune checkpoint antagonists in the treatment of breast cancer (37). Research shows that cellular progression, such as aging, affects the expression of PD-L1 at immune checkpoints (38), so that we speculated that whether MLN8237 could influence the immune response of the tumor by regulating PD-L1 expression while inhibiting the cell cycle. Here, we proved that MLN8237 alone reduced the volumes of transplanted tumors to some extent applied in the mouse breast cancer 4T1 (**Figure 5**).

However, as demonstrated by immunohistochemical analysis, CD3^+^ and CD8^+^T cells were less frequently observed in MLN8237-treated 4T1 tumor tissues, compared with the vehicle-treated group. The data indicated that MLN8237 could inhibit tumor growth to some extent. Meanwhile, MLN8237 also could impair T cell-mediated antitumor immune response through reducing the number of CD3^+^ and CD8^+^ T cells infiltrated *in vivo* with 4T1 mouse breast cancer cells (**Figure 5**), which exerted immunosuppressive effects. Furthermore, in order to better understand the effect of MLN8237 on anti-tumor immune response, we investigated the effect of MLN8237 on anti-tumor immune response using immunocompetent mice bearing MC38 tumor (39). Similarly, the suppression of p-AURKA induced by MLN8237 treatment could not significantly inhibit the growth of MC38 transplanted tumors. MLN8237 also significantly inhibited the infiltration of CD8^+^ T cells in the MC38 transplanted tumors (**Figure 6**).

In principle, MLN8237 is a cell cycle blocker, which may affect the infiltration of T cells in tumor tissue by inhibiting proliferation of T cells (40). Based on the tumor immunosuppression caused by decreased T-cell infiltration, we used anti-PD-L1 antibodies alone or in combination with MLN8237 to treat MC38 transplanted tumors. We found that anti-PD-L1 antibodies could significantly reverse the decrease of T cell infiltration caused by MLN8237 treatment (**Figure 6**). Above results suggested that MLN8237 inhibited the infiltration of T cells in tumor tissues, mainly by modulating the immune checkpoint PD-L1/PD-1. It showed that concurrent treatment with MLN8237 and immune checkpoint inhibitors significantly improved the therapeutic efficacy *in vivo* (**Figure 6**).

*In vitro*, our findings revealed that when the tumor cells were exposed to MLN8237, the upregulation of PD-L1 was more pronounced in 4T1 cells, which was coincident with the remarkable elevation of the PD-L1 in MC38 cells (**Figure 1F**). The study performed by ZHANG et al. demonstrates that PD-L1 is remarkably increased in the M/G1 phase compares to other cell cycle phases. When it is stimulated by mitosis signals, the cell cycle protein D (Cyclin D) interacts with CDK4 or CDK6 and then been activated. The blockade of SPOP phosphorylation by CDK4/6 inhibitors leads to the degradation of SPOP, which further restrains the ubiquitination-mediated PD-L1 degradation (20). It has reported that MLN8237 can induce G2/M cell phase arrest (41). Based on the findings in our study, the inhibition of cell cycle in G2/M phase induced by using AURKA inhibitor MLN8237 could also up-regulate the expression of PD-L1.

To explore the mechanism of MLN8237 promoting PD-L1 expression, we screened JAK/STAT, PI3K/AKT and MAPK/ERK signal paths with SKBR3 breast cancer cells, and the protein levels of p-STAT1, p-STAT3, p-STAT5, p-AKT and p-ERK were detected after treatment with MLN8237. The expression of p-STAT3 and p-AKT were upregulated in SKBR3 cells after treatment with MLN8237. Furthermore, WP1066 could reverse the upregulation of PD-L1 processed by MLN8237 by inhibiting p-STAT3 in breast cancer cells (**Figure 4**). Here, we put forward that the expression of PD-L1 could be upregulated by MLN8237-induced increase of p-STAT3. Given that STAT3 promotes breast cancer metastasis, it is also a potential target of breast cancer treatment (42). Our results suggested that MLN8237 and STAT3 inhibitors jointly treated breast cancer strategies may enhance antitumor immunity reaction by inhibiting tumor cell proliferation and metastasis.

## 5 Conclusion

MLN8237 has not been as effective in clinical trials. The effect of MLN8237 on the anti-tumor immune response is unclear. In this study, we found that MLN8237 can inhibit the anti-tumor immune response of T cells by upregulating PD-L1 of tumor cells. We have also made a preliminary analysis of its mechanism. We found that MLN8237 up-regulates PD-L1 by activating STAT3. Moreover, the combination of MLN8237 and anti-PD-L1 antibody could reverse the upregulation of PD-L1 and the decrease of CD8^+^ T cell tumor tissue infiltration induced by MLN8237 *in vivo*.

In conclusion, our study provides some clues for the poor efficacy of MLN8237 in clinical trials. We propose a new combination strategy, namely the combination of MLN8237 with immune checkpoint inhibitors, to enhance the therapeutic efficacy in tumor treatment. We have discovered a novel pathway of PD-L1 upregulation, which tumor cells may utilize to evade immune surveillance when their cell cycle processes are unexpectedly disrupted. It also can provide a huge reference value for PD-L1 in clinical optimal applications and offer a new possibility for a combination of targeted drug with immunotherapy.

## Data availability statement

The original contributions presented in the study are included in the article/**Supplementary Material**. Further inquiries can be directed to the corresponding authors.

## Ethics statement

The animal study was approved by Animal Care and Use Committee at Xuzhou Medical University. The study was conducted in accordance with the local legislation and institutional requirements.

## Author contributions 

HP, JZ and MS initiated, designed and supervised the study. BM, XZ and SJ performed the experiments. BM, XZ, ZX, SL, XW, WM, LL and DL wrote the paper. All authors have approved the manuscript. All authors have confirmed that they had full access to all the data in the study and accepted responsibility to submit for publication. All authors contributed to the article and approved the submitted version.
