# Supplementary material for: AURKA inhibitor-induced PD-L1 upregulation impairs antitumor immune responses
Source: Front Immunol. 2023 Sep 12;14:1182601. doi: 10.3389/fimmu.2023.1182601 (PMC10536236; doi:10.3389/fimmu.2023.1182601)
Supplement: Supplementary file 1 [file DataSheet_1.docx]

AURKA inhibitor-induced PD-L1 upregulation impairs antitumor immune responses

Bi Meng^1,2,3†^, Xuan Zhao^1,2,3†^, Shuchang Jiang^4^, Zijian Xu^1,2,3^, Sijin Li^1,2,3^, Xu Wang^1,2,3^, Wen Ma^1,2,3^, Liantao Li^2,3^, Dan Liu^1,2,3^, Junnian Zheng^2,3^*, Hui Peng^4^*, Ming Shi^1,2,3^*

1. Cancer Institute, Xuzhou Medical University, 209 Tongshan Road, Xuzhou, Jiangsu, 221004, China

2. Center of Clinical Oncology, The Affiliated Hospital of Xuzhou Medical University, 99 Huaihai Road, Xuzhou, Jiangsu, 221002, China

3. Jiangsu Center for the Collaboration and Innovation of Cancer Biotherapy, Xuzhou Medical University, 209 Tongshan Road, Xuzhou, Jiangsu, 221004, China

4. Department of Operational Medicine, Tianjin Institute of Environmental & Operational Medicine, Tianjin 300050, China

† or * These authors contributed equally to this work.

Correspondence to

Junnian Zheng, email: jnzheng@xzhmu.edu.cn.

Hui Peng, email: p_h2002@hotmail.com

Ming Shi, email: sm200@sohu.com

Supplement Figure：

Table 1 CCK-8 experiment detects IC_50_ of different tumor-targeted drugs

| **Cell** | **Drugs** | **Target** | **IC_50_ (**μM**)** |
| --- | --- | --- | --- |
| **SKBR3** | **ARN-509** | **Androgen Receptor** | **90** |
|  | **AZD3514** | **Androgen Receptor** | **55** |
|  | **Galeterone** | **Androgen Receptor** | **25** |
|  | **Neratinib** | **HER2** | **0.04** |
|  | **MLN8237** | **AURKA** | **6.5** |
|  | **LGK974** | **PORCN** | **12.5** |

Supplement Figure 1. IC_50_ of different tumor-targeted drugs was detected by CCK-8 experiment.


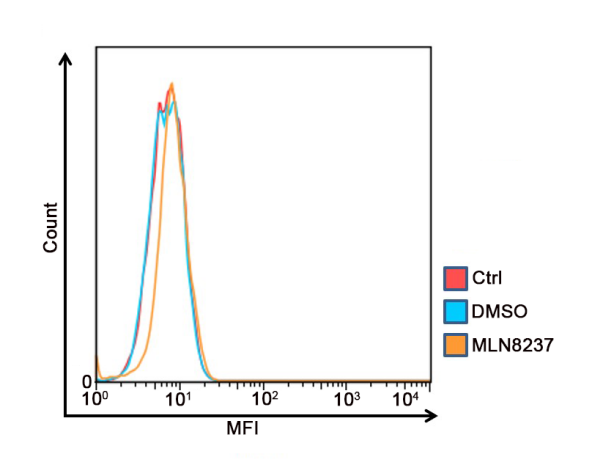


Supplement Figure 2. MLN8237 had no luminescent group. Detection the luminescent group of MLN8237 (0.8 μM) without secondary antibody.


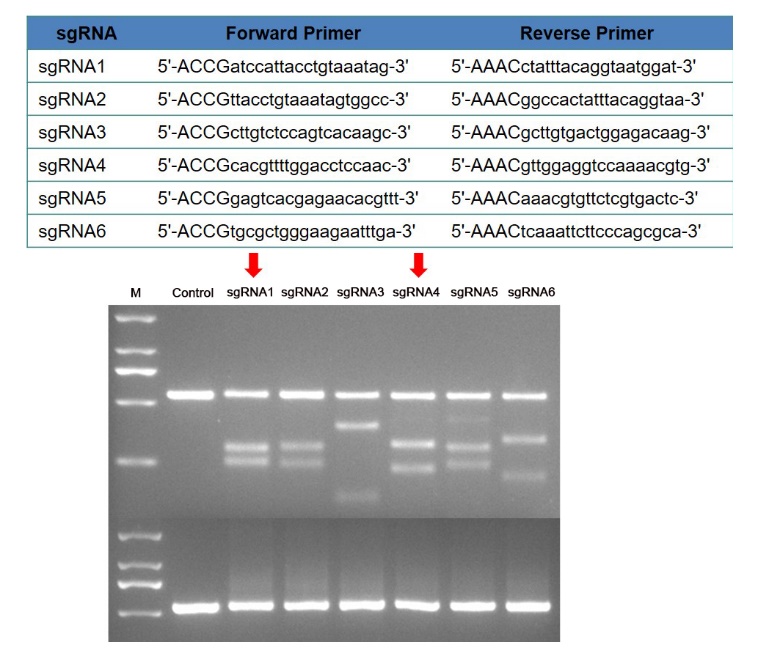


Supplement Figure 3. The cutting efficiency of sgRNA was detected. SKBR3 cells were transfected with Cas9-sgRNA1-sgRNA4 plasmid. Cells were obtained after 5 days of puromycin (1 μg/ml) drug screening, and then the cutting efficiency was calculated. PCR products were analyzed by using agarose gel electrophoresis (gel concentration 3%).
